# In defense of funding foundational plant science

**DOI:** 10.1093/plcell/koaf106

**Published:** 2025-05-05

**Authors:** Joanna D Friesner, Cristiana T Argueso, Wolfgang Busch, Thorsten Hamann, Lucia Strader, Mary Williams, Shuang Wu, Adrienne H K Roeder

**Affiliations:** North American Arabidopsis Steering Committee, Corvallis, OR 97330, USA; North American Arabidopsis Steering Committee, Corvallis, OR 97330, USA; Department of Agricultural Biology, Colorado State University, Fort Collins, CO 80523, USA; North American Arabidopsis Steering Committee, Corvallis, OR 97330, USA; Plant Molecular and Cellular Biology Laboratory, Salk Institute for Biological Studies, La Jolla, CA 92037, USA; Institute for Biology, Norwegian University of Science and Technology, Trondheim 7491, Norway; Multinational Arabidopsis Steering Committee; Department of Biology, Duke University, Durham, NC 27708, USA; American Society of Plant Biologists, Rockville, MD 20855, USA; State Key Laboratory of Agricultural and Forestry Biosecurity, College of Horticulture, Fujian Agriculture and Forestry University, Fuzhou 350002, China; North American Arabidopsis Steering Committee, Corvallis, OR 97330, USA; Weill Institute for Cell and Molecular Biology and School of Integrative Plant Science, Section of Plant Biology, Cornell University, Ithaca, NY 14853, USA

## Abstract

Plants are essential for life as we know it on Earth. They oxygenate the atmosphere, regulate the climate, and comprise much of the primary producers underpinning complex food systems. In the 1980s, a multinational group of plant scientists chose the small angiosperm—*Arabidopsis thaliana*—to serve as the model flowering plant for genetic and molecular studies that would be leveraged to produce vast new datasets, resources, and tools. The rationale they used to persuade funding agencies to make significant investments and focus intense effort on this single plant species was to produce a deep fundamental knowledge of the biology of plants and to apply this knowledge to valuable, but typically less tractable, plant species. Over the past 40 yr, Arabidopsis has emerged as the most powerful and versatile plant model to uncover core biological principles and served as a prototyping system to test advanced molecular and genetic concepts. We argue that the emerging challenges of accelerating climate instability and a rapidly growing global population call for renewed and robust investments in fundamental plant biology research. Leveraging the power of Arabidopsis research, resources, datasets, and global collaborative community is more important than ever. This commentary lays out a vigorous defense of foundational, i.e. “basic,” plant science research; describes that often, Arabidopsis is preferable to working directly in crops; highlights several transformative applications generated from basic plant research; and makes the argument that plant science is vital to the survival of humanity.

## Deep knowledge of plants is essential for our future: sustainable agriculture, climate change, and ecosystem services

Plants form the foundation of human civilization, providing numerous essential ecosystem services (e.g. oxygen via photosynthesis, aiding pollinators, regulating climate through carbon sequestration, mitigating soil erosion, and serving as a direct or indirect food source for all animal life) and enabling the production of food, feed, and fiber through agriculture. This has underpinned cultural achievements for millennia and sustains our modern societies. However, we are at a pivotal moment in history, facing 2 unprecedented challenges: accelerating environmental change and a rapidly growing global population (United Nations 2022; IPCC 2023). Climate instability is increasing at an unprecedented pace, with critical tipping points either already crossed or likely to be crossed within the lifetimes of a large fraction of this readership (McKay et al. 2022; IPCC 2023). Meanwhile, population projections estimate that the global human population will reach 10.3 billion people in the mid-2080s (United Nations 2024), requiring drastic changes in food consumption, production, management, and storage; otherwise, global agriculture must produce twice as much food as was produced at the beginning of the century. Even with significant changes in food management, agricultural production will need to increase substantially and will need to occur in a fundamentally altered environment. Whereas applied research is undoubtedly valuable in this pursuit, the fundamental discoveries made through basic plant biology research will lay the foundation for long-term, sustainable solutions.

By 2060 to 2080, an estimated 1.5 billion hectares of land previously unsuitable for agriculture are predicted to become available for cultivation, primarily in northern latitudes as climate zones shift (Franke et al. 2022). Concurrently, however, 2.2 billion hectares of land currently suitable for farming are projected to become less viable for agricultural production by 2071 to 2100, particularly in regions affected by desertification, extreme heat, salinization, sea level rise, or water scarcity (Hannah et al. 2020). At the same time, agriculture will face growing competition for land use, with increasing conflicts between food production and timber production, as rising temperatures and shifting precipitation patterns alter global forestry and agricultural landscapes (Bousfield et al. 2024).

How can we address these challenges? Much of our hope rests on the ability to engineer crops and optimize breeding strategies with far greater precision and speed than ever before. While the processes of breeding or gene editing, and the pipeline of testing and developing new crop varieties itself might not fundamentally change, there are crucial aspects that can be significantly accelerated by research in model species. This includes, foremost, a deep and reliable understanding of the biology of plants, gene function, and molecular pathways. In addition, model plants can serve for rapid prototyping of new technologies, as it is nearly impossible to develop such innovations directly in crops due to the high cost and time required for iterative cycles of designing, testing, and improving. Model plants can therefore enable not only the testing of engineering and breeding concepts but also the generation of essential data to support regulatory approval of novel traits and trait engineering approaches. Fundamental plant research, therefore, will continue to provide the foundation for agricultural innovation. Such innovation is needed to enable us to unravel complex genetic networks, develop climate-resilient crops, and accelerate breeding and genetic engineering efforts. For decades, *Arabidopsis thaliana* (“Arabidopsis”) has been the workhorse of plant biology, providing groundbreaking insights into plant gene function, cellular processes, and biological pathways (Provart et al. 2016; Yaschenko et al. 2024). Given current and future challenges, leveraging the power of Arabidopsis research is more vital than ever. The foundational discoveries made in Arabidopsis have allowed us to understand some of the most important plant processes, such as plant hormone signaling, stress responses, epigenetics, and plant architecture, laying the groundwork for applications in crop improvement, reforestation, and ecosystem management (Yaschenko et al. 2024). In addition, Arabidopsis research has contributed to our fundamental understanding of biological processes in humans. Most importantly, gene and pathway annotation, which is essential for genetic discovery and engineering in crops, is largely dependent on foundational research performed in Arabidopsis (Wimalanathan and Lawrence-Dill 2021; Yaschenko et al. 2024). Investing in Arabidopsis research is, therefore, not merely an academic pursuit to understand important biological processes that shape our (plant) world; it is an essential strategy for ensuring global food security, mitigating climate change impacts, and ensuring the sustainability of both agricultural and natural ecosystems.

## The role of Arabidopsis moving forward—Why not just work directly in crop plants?

The case for Arabidopsis as a genetically amenable organism was made strongly in the 1940s by Laibach (1943), due to its small size, short generation time, production of large numbers of progeny, and small chromosome number; these desirable genetic traits are augmented by its tendency to self-fertilize. As mutagenesis techniques were developed and improved, many mutants were generated (particularly auxotrophic mutants), which gave rise to information and seed-sharing communities (Rédei 1975). Interest in Arabidopsis continued at a slow but steady pace until plants entered their molecular era with the advent of molecular biology, and the invention of DNA isolation techniques that enabled phage library screening and gene cloning (see Somerville and Koornneef 2002). Interest was greatly renewed in Arabidopsis with the reporting that its small genome size enabled molecular approaches and highlighted the potential for map-based cloning; these advances rapidly led to the characterization of many genes initially identified through genetic studies (Meyerowitz 1987). In parallel, methods were developed to stably introduce foreign DNA into plant cells using *Agrobacterium tumefaciens*, further enhancing the molecular biologist's toolbox (Chilton et al. 1977). The elucidation of the complex pathway of homeotic genes controlling flower development demonstrated that Arabidopsis is also a good tool for developmental biologists (Coen and Meyerowitz 1991). The development of the floral dip technique greatly facilitated Agrobacterium-mediated transformation of Arabidopsis and led to the easy generation of plant mutants, thus rapidly facilitating genetic studies (Clough and Bent 1998). A further boost of Arabidopsis to model plant status came from coordinated funding by multinational science agencies of an ambitious collaborative project to sequence the Arabidopsis genome, and the landmark paper was published 25 yr ago by the Arabidopsis Genome Initiative (2000) on the “Analysis of the Genome Sequence of the Flowering Plant *Arabidopsis thaliana*.” This was the first plant genome sequenced and the third multicellular eukaryote, following *Caenorhabditis elegans* and *Drosophila melanogaster.*

Twenty-five years later Arabidopsis is arguably still the most powerful plant model organism and continues to underpin innovative plant research, as illustrated by many of the articles in this focus issue, as well as others (e.g. Provart et al. 2016; Yaschenko et al. 2024). However, with the sequencing of more plant genomes and the advent of genome editing technology that allows altering of gene function in other plant species, there has been a strategic shift away from prioritizing Arabidopsis research. Based on multiple lines of evidence, which we outline here, we argue that Arabidopsis remains a critically important research organism for the advancement of plant sciences and for solving important societal challenges of our time. First, all of the scientifically practical and attractive features identified by Laibach in the 1940s still hold true (Laibach, 1943). It is a small, rapidly developing plant that can be grown quickly and cheaply in small spaces; genetic lines can be readily propagated with little effort because it has a very high rate of self-fertilization, and its compact, diploid genome facilitates most genetic approaches and techniques, from GWAS to assay for transposase accessible chromatin sequencing. Additionally, the significant research investment over the past several decades means that for many loci, there are multiple mutant alleles available, augmented by the increasingly tractable natural genetic diversity of this species; these provide a wealth of information about gene and protein functions (Leventhal et al. 2025). Furthermore, as described (Roeder et al. 2025, this issue), the plant fortuitously has a floral structure that makes it almost uniquely amenable to transformation by the simple, fast floral dip method, facilitating gain-of-function studies, and closing the loop on hypothesis testing. Arabidopsis continues to be the premier angiosperm for fast, testable, reproducible research into fundamental plant processes.

It can be argued that Arabidopsis sits squarely as a hub for comparative studies across plant species. Its wealth of genetic resources and databases (e.g. The Arabidopsis Information Resource, www.arabidopsis.org) provides a starting point for comparative studies in any plant, whether another model species such as Marchantia polymorpha or a crop species (Provart et al. 2020). As artificial intelligence (AI)/machine learning is transforming the future of biological research, we speculate that the wealth of data in Arabidopsis is essential to train new AI models of many plant processes (Brady et al. 2025). Similarly, Arabidopsis is widely used for comparative studies beyond the plant kingdom, for example with yeast (*Saccharomyces cerevisiae*) or animal models (e.g. *D. melanogaster*, *C. elegans*) (Meyerowitz 2002; Nürnberger et al. 2004; Millar and Waterhouse 2005; Avin-Wittenberg et al. 2012). As compared with crops, studies using Arabidopsis are often less expensive, thus enabling larger experiments, such as assessing GxE (gene-environment) interactions on hundreds to thousands of genes in different genomic backgrounds, and across different environments. Unlike crops, Arabidopsis has not gone through severe domestication bottlenecks enabling evolutionary, ecological, and GxE research (1001 Genomes Consortium 2016; Gross and Olsen 2010; Platt et al. 2010). Additionally, the wealth of data from natural populations is without precedent; although many crops have recognized wild relatives (e.g. Glycine soja, teosinte, wild tomato), far fewer resources are available for comparative studies (see Arana and Pico 2025).

It is true that many of these advantages are not unique to Arabidopsis; for example, optimization of growth conditions both indoors and in the field can lead to rice generation times of just 2 months (Kabade et al. 2024; Sandhu et al. 2024). The *Nicotiana benthamiana* system is excellent for quick and easy gain-of-function studies through transient expression via agroinfiltration, but less amenable to classic genetic studies, and such studies are mainly limited to leaves (Ranawaka et al. 2023; Golubova et al. 2024). Likewise, somatic genetic transformation of hairy roots provides the ability to study gene function in crop species (Cheng et al. 2021) but is limited to roots in tissue culture. Additionally, new model systems have been developed for nonvascular bryophytes (*Physcomitrium patens* and *Marchantia polymorpha*) that are small and easy to manipulate in the laboratory, and that provide insights into land plant evolution.

As highlighted in one of the articles in this focus issue (Uauy et al. 2025), the plant kingdom is quite diverse, considering its recent origins, and perhaps there is no such thing as a “typical” plant. However, as an angiosperm with widespread geographic diversity, Arabidopsis ticks most of the “typical plant” boxes. In fact, most of the processes studied in Arabidopsis are found in most other plants (Yaschenko et al. 2024; Brady et al. 2025). Some conserved cellular processes include mechanisms of gene expression spanning chromatin, small RNAs, gene networks, transcription, translation, and protein transport and processes. Conserved physiological processes include primary plant metabolism, photosynthesis and (C3) carbon fixation, and the responses to biotic and abiotic conditions. Developmental processes, from cell division and patterning to gene regulatory networks and morphogens, are also largely conserved, at least in principle. Nevertheless, no single model species can represent all plants. Not only does Arabidopsis lack the otherwise widely abundant mycorrhizal associations, it is also not suitable for studying C4 or CAM photosynthesis, symbiotic nitrogen fixation, perennial growth and wood formation, fleshy fruit development, or monocot-specific traits (see also Roeder et al. 2025).

The strong global community of Arabidopsis researchers, whose genesis was the multinational coordinated Arabidopsis genome sequencing project in the early 1990s, is sustained via the International Conference on Arabidopsis Research (ICAR), which rotates between North America, Asia/Pacific Rim, and Europe and is organized by local community volunteers. ICAR is the premier annual international conference for Arabidopsis researchers and attracts ∼500 to 1,500 participants annually, including non-Arabidopsis researchers, due to its reputation for featuring cutting-edge research and for facilitating interactions between scientists of all career stages. The success of this research field has been greatly facilitated by ICAR, the major stock centers that distribute seed and DNA resources, the annotated genome sequences, and the sharing of community-generated datasets, tools, techniques, and resources. Finally, we should point out that Arabidopsis has proven to be a highly accessible plant for teaching and training at both the K-12 school and college levels (Woodward and Bartel 2018). For example, the ABRC and NASC stock centers distribute hundreds of educational seed stocks to schools every year, as well as educational kits with seeds and protocols that were developed by, and in collaboration with, researchers (https://abrc.osu.edu/educators/education). Arabidopsis database resources and wealth of publications and seed stocks provide students with an unparalleled, authentic exposure to plant molecular biology research.

## Need for basic research in all plants, including crops

For decades, Arabidopsis has served as a bedrock model system in plant biology, providing a simplified yet powerful platform to dissect evolutionarily conserved pathways with genetic precision. However, mounting pressures to address climate change, population growth, and soil degradation have increasingly redirected plant biology toward applied research in crops and species of immediate agronomic value. While this practical shift is important, it risks undervaluing the foundational role of curiosity-driven basic science. History repeatedly demonstrates that transformative agricultural breakthroughs are often rooted in fundamental discoveries. As highlighted by the National Research Council, over 70% of agricultural advancements trace back to research in model organisms or noncrop species (National Research Council 2008). Simultaneously, after 40 yr of research in the Arabidopsis model system, we appreciate how limited our mechanistic understanding still is. For 70% of predicted Arabidopsis genes, we still need to confirm experimentally their Gene Ontology Molecular Function or Biological Process annotations (Gene Ontology Consortium 2010), and the ability to functionally annotate crop genes largely depends on such functional data from Arabidopsis.

Genes identified in Arabidopsis, such as homologs of FLOWERING LOCUS T, have enabled breeders to tailor photoperiod-sensitive crops such as rice and soybean to diverse latitudes and climate zones (Xue et al. 2008; Jung and Müller 2009). Beyond genes, methodologies such as multiomics approaches, now integral to molecular breeding, were also first pioneered in model species (Rhee and Mutwil 2014). Yet long before Arabidopsis became prominent, foundational discoveries in diverse plant species laid the groundwork for agricultural revolutions. Darwin's 1880s experiments on phototropism in canary grass (*Phalaris canariensis*) and subsequent auxin isolation in oat coleoptiles revolutionized our understanding of plant growth regulation (Darwin and Darwin 1880; Went 1928). Deciphering auxin's roles in development and stress adaptation later yielded tools ranging from synthetic herbicides to techniques for optimizing fruit set under suboptimal pollination conditions (e.g. auxin-induced parthenocarpy in greenhouse tomatoes) (Gustafson 1936; Grossmann 2010). Similarly, the Green Revolution, which reshaped global agriculture, was built on decades of basic studies on stem elongation, plant architecture, and gibberellin responses (Peng et al. 1999). These examples reveal a common pattern: applied solutions often begin with fundamental discoveries arising from curiosity-driven research. Even for discoveries initially made in crops—like Barbara McClintock's identification of the first transposable element in maize—later research elucidating how these elements function, how they are regulated, and their role in genome evolution and biotechnology was largely conducted in *Arabidopsis*.

Advances in genetic and transformation techniques now enable crops themselves to serve as platforms for basic research. In recent years, more and more translation of basic research has been performed directly in crop species. In tomato, studies on genetic redundancy, gene dosage, and epistasis in development, evolution, and domestication have redefined innovative strategies for crop improvement (Soyk et al. 2017; Hendelman et al. 2021; Aguirre et al. 2023). Tomato plants are strictly self-pollinating, a process enabled by cleistogamous flowers that remain stigma unexposed during pollination. A recent study identified HD-Zip genes as the key regulators of the anther cone formation critical for tomato's self-pollination (Wu et al. 2024). In rice, the tissue-specific brassinosteroid signaling has been shown to fine-tune the balance between panicle branching and grain size—a discovery with direct yield implications (Zhang et al. 2024). Maize research has unveiled a HY5–COOL1–CPK17 regulatory module integrating light signaling and calcium-dependent kinase activity in maize cold tolerance, offering clues for adapting crops to higher latitudes (Zeng et al. 2025).

Crop-centered studies also address specialized traits that are absent in Arabidopsis. Tomato serves as a model for fruit ripening, providing insights into postharvest quality, while rice and Medicago have advanced our understanding of plant–microbe interactions, such as arbuscular mycorrhizal symbiosis and nitrogen fixation—knowledge critical for engineering sustainable cereals (Giovannoni 2004; Parniske 2008). Similarly, research on C4 photosynthesis in maize or salt-secreting quinoa explores evolutionary adaptations that could inspire climate-resilient crops (Langdale 2011; Adolf et al. 2013). These systems remind us that nature's diversity often holds untapped solutions for pressing agricultural challenges. While these are excellent examples of questions that can only be addressed in specific plants or crops, studying many conserved fundamental processes in such systems is often less practical. For instance, investigating highly conserved processes such as transcription, splicing, DNA repair, or translation in a complex crop system rather than in Arabidopsis would likely be more expensive, time-consuming, and even impractical due to limited tools and resources. Ultimately, the answers obtained would probably be the same as those achievable more efficiently using the model organism.

In the pursuit of addressing the challenges of the future, we must remember that today's applied breakthroughs often emerge from yesterday's fundamental discoveries. Arabidopsis remains indispensable for foundational insights, similar to *D. melanogaster* and *C. elegans*. However, the next frontier of plant biology lies in bridging model systems and agriculture through translational research in crops and ecologically unique species. By closely integrating curiosity-driven science in crops and other nonmodel plant species with traditional model species such as Arabidopsis, we ensure that the seeds of basic discovery continue to bear fruit for future agricultural innovations.

## Transformative applications arise from fundamental research

With so many dire and pressing problems to be solved, it could be argued that scientists should direct all research efforts into addressing these problems. Many scientists are doing just that. However, a singular focus on solving today's problems would be a mistake. We argue that in addition to research that has an obvious direct application, it is vital for scientists to continue researching fundamental plant science questions, for which the applications may not currently be obvious, and whose full value may be realized decades in the future. The importance of basic research to breakthrough applications was described by Dr. Laurie Glimcher, M.D., President and CEO of the Dana-Farber Cancer Institute and Richard and Susan Smith Professor of Medicine at Harvard Medical School: “There is a famous story of a drunk looking for his lost keys under a streetlight because the light is better there … if we only look for cures where the light has already shone, we will make few, if any, new discoveries. Basic research shines a light into the dark corners of our understanding, and by that light we can find wonderful new things” (Bergman 2018). This principle applies equally to improving agriculture as it does to medicine.

Although fundamental research is often spurred by an element of curiosity, generally these research projects are done with the idea of some eventual potential application in mind. However, the most transformative and impactful applications are often those that were not foreseen at the time the research was started. Many such breakthroughs open up new avenues that were not even contemplated before the discovery. Often such breakthroughs are built on decades of basic discoveries from several different labs working on different angles of a question and exchanging information at scientific conferences and meetings. For example, CRISPR was first detected as a puzzling repeat sequence in the *Escherichia coli* genome by scientists studying the gene responsible for isozyme conversion of alkaline phosphatase in 1987; at the time it was not possible to predict their biological function (Ishino et al. 1987, 2018). Even after the biological function of CRISPR in bacterial immunity against viruses was discovered by 3 labs in 2005 (Bolotin et al. 2005; Mojica et al. 2005; Pourcel et al. 2005; Lander 2016), it took 3 yr for the speculation that CRISPR could be used for genome editing in a heterologous system (Marraffini and Sontheimer 2008). The motivation of the scientists who contributed these early discoveries was understanding repeat sequences in salt-tolerant microbes, defending against biological warfare, and improving yogurt production, not editing genomes (Lander 2016). From 2008 on, many other scientists and additional basic research discoveries were involved in developing the revolutionary genetic engineering technology introduced in 2012 and 2013 that has transformed nearly every area of biology from basic research to applications in engineering crop plants and human medicine (Lander 2016; Ledford 2016; Zhu et al. 2020).

Of the 28 most transformative medicines that the FDA approved between 1985 and 2009, 80% could be traced to a basic research discovery (Spector et al. 2018). The first basic research discovery leading to these drugs occurred an average of 31 yr before FDA approval, long before any druggable target was identified (Spector et al. 2018). Fundamental research in Arabidopsis has already contributed to our understanding of human health. 70% of human cancer genes have a related gene in Arabidopsis (Jones et al. 2008). For example, the COP9 signalosome was first discovered and studied in Arabidopsis, which contributed to understanding regulation of the P53 tumor suppressor gene in mammals (Dornan et al. 2004; Jones et al. 2008). Similarly, research on the NLR receptors in plants has contributed to advancing our understanding of the innate immune system in humans and its contribution to autoimmune diseases such as Crohn's (Jones et al. 2016; Chou et al. 2023). Likewise, the blue light receptor (CRY1) was first found in plants and later the related gene was found in mammals (Thresher et al. 1998; Jones et al. 2008). CRY1 regulates circadian rhythm and a mutation in human CRY1 leads to sleep disruption and “night owl” behavior (Patke et al. 2017). Further fundamental work on how plants sense light in Arabidopsis, algae, and other plant species led to the identification of the molecular mechanisms of light receptors (Möglich et al. 2010; Galvão and Fankhauser 2015). This basic research led to the development of optogenetic tools in which a scientist or clinician can use light to turn various proteins on and off (Emiliani et al. 2022). Optogenetics has been employed for research in animals and plants and is just beginning to be employed in human medicine; optogenetic therapies are being developed now to treat diabetes, heart disease, and cancer (Ye and Fussenegger 2019; Yaschenko et al. 2024).

Likewise, basic plant science research leads to unanticipated applications in agriculture. For example, decades of research on glucosinolates and the myrosinase enzymes that break them down in Arabidopsis and other Brassicaceae plants (Halkier and Gershenzon 2006) was the basis for Pairwise to develop better tasting, nutritious Conscious Greens. Pairwise used CRISPR to knock out the myrosinase genes in mustard greens, making them less bitter, so that consumers will eat these highly nutritious leaves in salads instead of less nutritious lettuce (Grinstein 2023). Similarly, using extensive knowledge from Arabidopsis research, pennycress (*Thlaspi arvense*), a closely related brassica, has been rapidly domesticated through breeding and genomics-enabled mutagenesis into a winter annual oilseed crop marketed by CoverCress (Chopra et. al. 2018, 2020; Phippen et al. 2022). CoverCress can be grown in corn and soy fields between crops in the winter acting as a cover crop to reduce nutrient leaching and soil erosion. The harvested oil can be used for bioenergy and the remaining meal can be used for animal feed, making it economically viable for farmers. In addition, research on development of the Arabidopsis fruit led to the discovery of the *INDEHISCENT* gene which controls pod shatter, the opening of the seed pod to disperse the seeds (Lilijegren et al. 2004). Seedpod shatter causes canola farmers to lose substantial yield when the valuable seeds fall, contaminating the field for the next growing cycle. Using this knowledge from Arabidopsis, BAYER developed the PodGuard trait in canola, which is now distributed to canola farmers by BASF in all of their hybrid seeds (Liljegren and Yanofsky 2006; Vancanneyt et al. 2010; Aguilera 2019). Farmers have said this is “game-changing” technology because it: (i) increases yield at harvest; (ii) allows them to leave the canola standing in the field (because they will not lose seed) while they harvest other crops such as soybeans, thus giving harvest flexibility; and (iii) it protects them against the huge seed losses that often occur if high winds of hail storms occur near the time of harvest.

In another example, there was no obvious agricultural application when Arabidopsis researchers began investigating how the shoot apical meristem maintains its size throughout development despite constantly losing cells into organ primordia. They identified the CLAVATA-WUSCHEL feedback loop as the mechanism that maintains this balance (Fletcher et al. 1999; Schoof et al. 2000; Yadav et al. 2011). Since then, CLAVATA signaling has been found to be widely conserved for the maintenance of proper meristem size from the moss *Physcometrium patens* to tomatoes and maize (Xu et al. 2015; Somssich et al. 2016; Whitewoods et al. 2018). In tomatoes, fruit size is a consequence of floral meristem size, which is regulated by the CLAVATA pathway. During domestication, fruit size increased largely due to QTLs at 2 loci: a promoter inversion in the tomato *CLAVATA3*, and mutations in a regulatory site in the 3′UTR of tomato *WUSCHEL* (Muños et al. 2011; Xu et al. 2015; Bennett et al. 2025). In maize, mutations in the *CLAVATA* pathway increase the number of kernel rows around the ear, suggesting that mild decreases in CLAVATA signaling may increase yield (Je et al. 2016). Now, CRISPR promoter mutations are being deployed to modify CLAVATA signaling to select beneficial traits such as increased yield (Liu et al. 2021). Thus, fundamental research on the maintenance of the meristem in Arabidopsis is having practical applications on yield in many crop plants.

## Because plants are primary to human life, it is essential that we humans understand them

In the face of mounting pressure from climate change, food insecurity, and population growth, increased investment in plant science research will be essential to meet future food production needs. Climate change affects not only plant growth, but also plant disease and insect pressure, considerably reducing crop yield, pre- and postharvest (Deutsch et al. 2018; Chaloner et al. 2021; Gerken and Morrison III 2022). Climatic changes also negatively affect agricultural practices and food distribution systems, significantly disrupting food security (Bezner Kerr et al. 2022). Thus, the need to understand the fundamentals of plant biology has never been more urgent.

Research in Arabidopsis can lead plant sciences in the discovery of new strategies for efficient carbon capture, as well as ways to engineer plants with climatic resilience, helping to significantly reduce the effects of climate change in agricultural and natural ecosystems. Investment in basic research fuels new ideas to transform translational approaches. Further, investment in plant science research will also be essential for the restoration of ecosystems, soils, and degraded environments, which are expected to increase due to intensified anthropogenic activity.

Over the past decade, research on Arabidopsis has been progressively deprioritized by major US funding agencies, including the Department of Energy, the US Department of Agriculture's National Institute of Food and Agriculture, and the National Science Foundation (NSF), particularly within the Plant Genome Research Program. In some cases, proposals centered on Arabidopsis are no longer eligible for submission. Given the foundational role of Arabidopsis in advancing plant biology and serving as a critical training platform for emerging scientists, this shift in funding priorities has led to a marked decline in both scientific advancement within the field and the development of the next generation of plant researchers. We therefore strongly urge the United States and global funding agencies to strengthen and sustain robust funding for Arabidopsis and fundamental plant research.

Due to the magnitude of the challenges ahead, collaboration between plant scientists of all countries will therefore be more important than ever. Given the unequal access to scientific, genetic, and personnel resources in different parts of the world, significant progress will only happen through the combined effort of scientific communities, and the exchange of knowledge. Rather than competition amongst research groups, collaboration and data sharing should be the model, to allow for faster progress on the significant challenges facing our societies. This shift is especially urgent in light of a troubling global trend: the rise of antiscience sentiment and the reduction of public funding for research in many countries. These developments threaten to erode scientific capacity at precisely the moment when it is most needed. Mitigating this will require new strategies, perhaps including a significant change in the way scientists are rewarded for their efforts, moving from a system of recognition for high-impact publications, to recognition for scientific collaborations, societal and environmental impact (Allen 2025), a change that would have to be adopted and fostered by funding agencies, universities and scientific societies.

Together, we issue a call to all plant scientists and citizens to defend investment in Arabidopsis and fundamental plant science research worldwide through outreach to funding agencies, scientific societies and governments, and the education of the general public about the importance of plants and plant science research to secure a healthy and sustainable future for our civilization and our planet.

## Data Availability

No new data were generated or analyzed in support of this commentary.

## Dive Curated Terms

The following phenotypic, genotypic, and functional terms are of significance to the work described in this paper:

CRY1 Gramene: ostta15g0100
CRY1 Araport: ostta15g0100

COP9 Gramene: AT4G14110
COP9 Araport: AT4G14110

CLAVATA3 Gramene: AT2G27250
CLAVATA3 Araport: AT2G27250

WUSCHEL Gramene: AT2G17950
WUSCHEL Araport: AT2G17950

INDEHISCENT Gramene: AT4G00120
INDEHISCENT Araport: AT4G00120

FT Gramene: AT1G65480
FT Araport: AT1G65480
